# Association between hospital case volume and mortality in pediatric sepsis: A retrospective observational study using a Japanese nationwide inpatient database

**DOI:** 10.2478/jccm-2025-0006

**Published:** 2025-01-31

**Authors:** Shingo Ohki, Makoto Otani, Shinichi Tomioka, Kosaku Komiya, Hideki Kawamura, Taka-aki Nakada, Satoshi Nakagawa, Shinya Matsuda, Nobuaki Shime

**Affiliations:** Graduate School of Biomedical and Health Sciences, Hiroshima University, Hiroshima, Japan; Shonan Kamakura General Hospital, Kamakura, Japan; University of Occupational and Environmental Health, Kitakyushu, Japan; Kotonoha Collaboration Clinic, Kitakyushu, Japan; Faculty of Medicine, Oita University, Oita, Japan; Kagoshima University Hospital, Kagoshima, Japan; Graduate School of Medicine, Chiba University, Chiba, Japan; National Center for Child Health and Development, Tokyo, Japan

**Keywords:** sepsis, pediatrics, hospital case volume, volume-outcome relationship, propensity score

## Abstract

**Introduction:**

The survival benefits of treatment at high-volume hospitals (HVHs) are well-documented for several critical pediatric conditions. However, their impact on pediatric sepsis, a leading cause of mortality among children, remains understudied.

**Aim of the study:**

To investigate the association between hospital case volume and mortality rates in pediatric sepsis.

**Material and Methods:**

We conducted a retrospective cohort study using data from the Diagnosis Procedure Combination database. The study included patients who met the following criteria: 1) aged 28 days to 17 years; 2) discharged from the hospital between April 2014 and March 2018; 3) had a sepsis diagnosis coded under the International Classification of Diseases, 10th revision; 4) underwent blood cultures on hospital admission day (day 0) or day 1; 5) received antimicrobial agents on day 0 or 1; and 6) required at least one organ support measure (e.g., mechanical ventilation or vasopressors) on day 0 or 1. Hospitals were categorized by case volume during the study period, with HVHs defined as those in the highest quartile and low-volume hospitals (LVHs) as those in the remaining quartiles. In-hospital mortality rates between HVH and LVH groups were compared using mixed-effects logistic regression analysis with propensity score (PS) matching.

**Results:**

A total of 934 pediatric patients were included in the study, with an overall in-hospital mortality rate of 16.1%. Of them, 234 were treated at 5 HVHs (≥26 patients in 4 years), and 700 at 234 LVHs (<26 patients in 4 years). Upon PS matching, patients treated at HVHs demonstrated significantly lower odds of in-hospital mortality compared with those treated at LVHs (odds ratio, 0.42; 95% confidence interval, 0.22–0.80; P = 0.008).

**Conclusions:**

In pediatric patients with sepsis, treatment at HVHs was associated with lower odds of in-hospital mortality.

## Introduction

Sepsis is associated with a high mortality rate and is one of the most life-threatening diseases in children [1,2]. Recognizing the global burden, the World Health Organization has recently highlighted the importance of improving sepsis management [3].

Previous studies have demonstrated that treatment at high-volume hospitals (HVHs) is associated with improved outcomes in critically ill patients. This relationship, known as the “volume–outcome” relationship [4], has been observed in several critical conditions among pediatric patients, such as cardiopulmonary arrest, fulminant myocarditis, and severe trauma [5,6,7]. While the survival benefits of treatment at HVHs for sepsis are well-established in adults [8,9], they have not been extensively studied in children.

Understanding the effect of hospital case volume on pediatric sepsis is crucial. Comprehensive approaches, such as enhancing initial management at low-volume hospitals (LVHs) and centralizing critically ill septic patients to HVHs, could potentially improve outcomes.

Consequently, this study aimed to evaluate the association between hospital case volume and mortality in pediatric sepsis.

## Materials and methods

### Study design and data source

This nationwide retrospective observational study was conducted in Japan between April 2014 and March 2018. Data from the Diagnosis Procedure Combination (DPC) database, a Japanese nationwide inpatient registry, was used. Details of the DPC database have been described previously [10,11]. The following information is available from the DPC database [6,11]: baseline patient characteristics (e.g., age at admission, sex, referral from another hospital, and transport by ambulance); diagnoses recorded using both International Classification of Diseases, 10th revision (ICD-10) codes and Japanese texts; dates of medication use, medical procedures, and medical devices; discharge status (discharge to home, discharge to a nursing facility, transfer to another hospital, or death in hospital); hospital length of stay; and total medical costs. The DPC database categorizes diagnoses as follows [12]: main diagnosis, admission-precipitating diagnosis, most resource-consuming diagnosis, second-most resource-consuming diagnosis, comorbidities present at admission, and conditions arising after admission. During the study period, data from >1,000 acute care hospitals, including 80 university and 16 children's hospitals, were included in the DPC database. The database covered >90% of tertiary care emergency hospitals in Japan [6]. According to a previous validation study, the sensitivity and specificity of the main diagnosis (defined as main diagnosis or admission-precipitating diagnosis) in the DPC database were 78.9% and 93.2%, respectively [13].

This study was approved by the Institutional Review Board of Hiroshima University (approval number: E2020-2332; study title: evaluation of the volume–outcome relationship in pediatric sepsis using the DPC database; approval date: January 1, 2021). Database access was granted by the University of Occupational and Environmental Health. All procedures adhered to the institutional ethical standards on human experimentation and with the Declaration of Helsinki established in 1975. The requirement for informed consent was waived due to the study's retrospective nature and the use of anonymized patient data in the DPC database.

### Patient selection

This study evaluated patients who met the following inclusion criteria: 1) aged 28 days to 17 years; 2) discharged from the hospital between April 2014 and March 2018; 3) had a sepsis diagnosis coded under the International Classification of Diseases, 10th revision; 4) underwent blood cultures on hospital admission day (day 0) or day 1; 5) received antimicrobial agents on day 0 or 1; and 6) required at least one of the following organ support measures on day 0 or 1: mechanical ventilation, inotropes/vasopressors (norepinephrine, epinephrine, dopamine, dobutamine, milrinone, olprinone, and vasopressin), cardiopulmonary resuscitation (CPR), mechanical circulatory support (MCS) (intra-aortic balloon pump or extracorporeal membrane oxygenation), and blood purification (intermittent or continuous renal replacement therapy, hemoadsorption, and plasma exchange) [14,15]. The ICD-10 codes for sepsis are listed in **Table 1**. Patients with missing data on referral from another hospital, date of diagnostic test or treatment, hospital discharge status, or hospital identification code were excluded.

**Table 1.** List of International Classification of Diseases, 10th Revision codes for sepsis

**Code**	**Diseases**
A02.1	Salmonella sepsis
A20.7	Septicaemic plague
A22.7	Anthrax sepsis
A26.7	Erysipelothrix sepsis
A32.7	Listerial sepsis
A39.2	Acute meningococcaemia
A39.3	Chronic meningococcaemia
A39.4	Meningococcaemia, unspecified
A40.0	Sepsis due to streptococcus, group A
A40.1	Sepsis due to streptococcus, group B
A40.2	Sepsis due to streptococcus, group D
A40.3	Sepsis due to Streptococcus pneumoniae
A40.8	Other streptococcal sepsis
A40.9	Streptococcal sepsis, unspecified
A41.0	Sepsis due to Staphylococcus aureus
A41.1	Sepsis due to other specified staphylococcus
A41.2	Sepsis due to unspecified staphylococcus
A41.3	Sepsis due to Haemophilus influenzae
A41.4	Sepsis due to anaerobes
A41.5	Sepsis due to other gram-negative organisms
A41.8	Other specified sepsis
A41.9	Sepsis, unspecified
A42.7	Actinomycotic sepsis
B37.7	Candidal sepsis
O85	Puerperal sepsis
P36.0	Sepsis of newborn due to streptococcus, group B
P36.1	Sepsis of newborn due to other and unspecified streptococci
P36.2	Sepsis of newborn due to Staphylococcus aureus
P36.3	Sepsis of newborn due to other and unspecified staphylococci
P36.4	Sepsis of newborn due to Escherichia coli
P36.5	Sepsis of newborn due to anaerobes
P36.8	Other bacterial sepsis of newborn
P36.9	Bacterial sepsis of newborn, unspecified

### Hospital categorization

We categorized hospitals by case volume during the study period. HVHs were defined as those in the highest quartile, while low-volume hospitals (LVHs) were those in the remaining quartiles.

### Patient variables and outcome

The following variables were extracted from the DPC database: age at hospital day 0; sex; referral from another hospital; transport by ambulance; diagnosis; diagnostic test or treatment on day 0 or 1 (blood cultures, antimicrobial agents, mechanical ventilation, inotropes/vasopressors, CPR, MCS, blood purification, blood transfusion, intravenous immunoglobulin, albumin, recombinant thrombomodulin, antithrombin, corticosteroids [hydrocortisone, methylprednisolone, prednisolone, dexamethasone, and betamethasone], and enteral nutrition); hospital discharge status (dead or alive); and hospital identification code. In-hospital mortality rates were compared between patients treated at HVHs (HVH group) and those treated at LVHs (LVH group).

### Statistical analysis

Qualitative variables were expressed as frequencies and percentages. Quantitative variables were reported as means and standard deviations (SDs). We initially planned to use multiple imputation to address potential missing values. However, the final dataset was complete, with no missing data. Consequently, multiple imputation was unnecessary [16,17]. In the unmatched cohort, crude in-hospital mortality rates of the HVH and LVH groups were compared using Fisher's exact test.

Propensity score (PS) matching was used in the main analysis to balance the potential confounders between the HVH and LVH groups [18]. The PS for being treated at HVHs was calculated using a multivariable logistic regression model. To adjust for organ failure status, already-known prognostic factors in pediatric sepsis, and factors of our interest, the following were selected as independent variables for the PS model [19,20,21,22,23]: age at hospital day 0, sex, referral from another hospital, transport by ambulance, and treatment on day 0 or 1 (mechanical ventilation, inotropes/vasopressors, CPR, MCS, blood purification, blood transfusion, intravenous immunoglobulin, albumin, recombinant thrombomodulin, antithrombin, corticosteroids, and enteral nutrition). The discriminative ability of the PS model was evaluated using the C-statistic.

Thereafter, one-to-one nearest-neighbor matching was performed without replacement. The caliper width of the pooled PSs was set to 0.2 SD. Variables were compared before and after PS matching, and those with an absolute standardized mean difference of <0.1 were considered well-balanced. Additionally, Kernel density plots for the distribution of the PS were created before and after matching. The odds of in-hospital mortality were compared using a mixed-effects logistic regression analysis, accounting for patient clustering within each hospital.

For sensitivity analyses, the following mixed-effects logistic regression analyses were performed: 1) an analysis with inverse probability of treatment weighting (IPTW) [18], 2) an analysis with PS matching excluding patients referred from another hospital, and 3) an analysis with PS matching excluding patients who received CPR on day 0 or 1.

All statistical tests were two-sided, and P values <0.05 were considered statistically significant. Stata/MP 15 (Stata Corp. 2017. Stata Statistical Software: Release 15. College Station, TX, StataCorp LLC) was used for all statistical analyses.

## Results

**Fig. 1.**
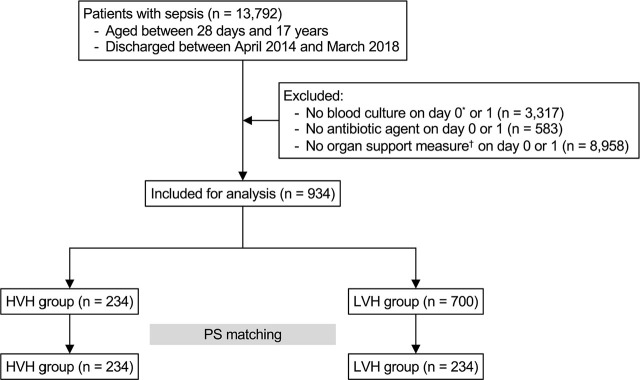
**Flow diagram of the study.** HVH, high-volume hospital; LVH, low-volume hospital; PS, propensity score. ^*^ Day 0 represents the hospital admission day. ^†^ Organ support measures include the following: mechanical ventilation, inotropes/vasopressors, cardiopulmonary resuscitation, mechanical circulatory support, and blood purification.

In total, 934 pediatric patients with sepsis were included in this study (Figure 1). The mean age was 4.7 years (SD, 5.3), 520 (55.7%) were male, and the overall in-hospital mortality rate was 16.1%. Among the 934 patients, 234 were treated at HVHs (five hospitals; ≥26 patients in 4 years), and 700 at LVHs (234 hospitals; <26 patients in 4 years). A comparison of patient characteristics and treatments between the HVH and LVH groups in the unmatched cohort is shown in Table 2. The crude in-hospital mortality rate was significantly lower in the HVH group than in the LVH group (8.6% vs. 18.6%; P < 0.001).

**Table 2.** Patient characteristics and treatment before and after PS matching

**Variable**	**Unmatched cohort (n=934)**			**Matched cohort (n=468)**		

	**HVH group (n=234)**	**LVH group (n=700)**	**ASMD**	**HVH group (n=234)**	**LVH group (n=234)**	**ASMD**
Age (years)	3.1 (4.3)	5.2 (5.6)	0.438	3.1 (4.3)	3.1 (4.5)	0.011
Male	118 (50.4)	402 (57.4)	0.141	118 (50.4)	112 (47.9)	0.051
Referral from another hospital	118 (50.4)	403 (57.6)	0.144	118 (50.4)	100 (42.7)	0.154
Transport by ambulance	144 (61.5)	355 (50.7)	0.219	144 (61.5)	141 (60.3)	0.026
Treatment on day 0^*^ or 1						
Mechanical ventilation	218 (93.2)	612 (87.4)	0.194	218 (93.2)	214 (91.5)	0.064
Cardiopulmonary resuscitation	12 (5.1)	61 (8.7)	0.141	12 (5.1)	13 (5.6)	0.019
Mechanical circulatory support	6 (2.6)	12 (1.7)	0.059	6 (2.6)	5 (2.1)	0.028
Inotropes/vasopressors	122 (52.1)	373 (53.3)	0.023	122 (52.1)	125 (53.4)	0.026
Blood purification	14 (6.0)	62 (8.9)	0.110	14 (6.0)	12 (5.1)	0.037
Blood transfusion	87 (37.2)	233 (33.3)	0.081	87 (37.2)	85 (36.3)	0.018
Intravenous immunoglobulin	42 (18.0)	204 (29.1)	0.266	42 (18.0)	40 (17.1)	0.022
Albumin	69 (29.5)	170 (24.3)	0.117	69 (29.5)	66 (28.2)	0.028
Recombinant thrombomodulin	13 (5.6)	127 (18.1)	0.397	13 (5.6)	15 (6.4)	0.036
Antithrombin	24 (10.3)	122 (17.4)	0.209	24 (10.3)	27 (11.5)	0.041
Corticosteroids	86 (36.8)	284 (40.6)	0.078	86 (36.8)	88 (37.6)	0.018
Enteral nutrition	103 (44.0)	154 (22.0)	0.481	103 (44.0)	92 (39.3)	0.095

Values are given as n (%) or mean (standardized deviation). HVH, high-volume hospital; LVH, low-volume hospital; ASMD, absolute standardized mean difference.

* Day 0 represents the hospital admission day.

### Main analysis

**Fig. 2.**
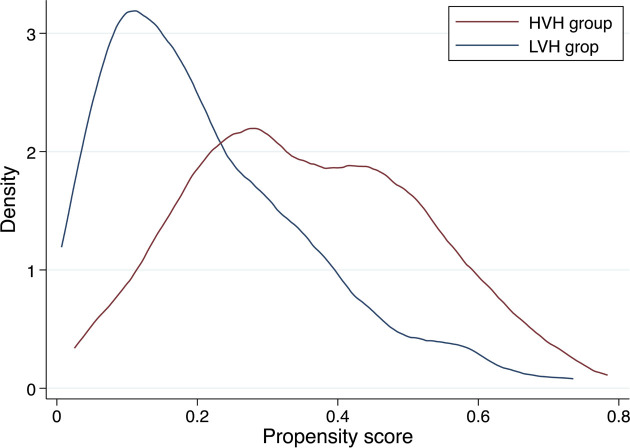
**Distribution of the propensity score before matching.** HVH, high-volume hospital: LVH, low-volume hospital.

**Fig. 3.**
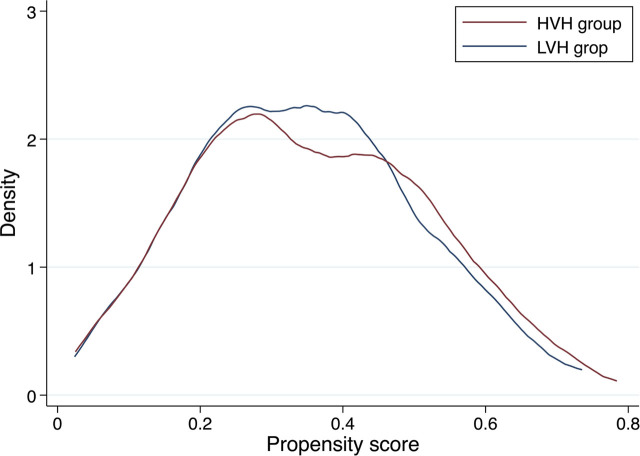
**Distribution of the propensity score after matching.** HVH, high-volume hospital: LVH, low-volume hospital.

The C-statistic for the PS model was 0.75. After PS matching between the HVH and LVH groups, 234 pairs of patients were included for further analysis. In the PS-matched cohort, the absolute standardized mean differences of all variables except for referral from another hospital were <0.1 and well-balanced between the groups (Table 2). Kernel density plots for the distribution of the PS before and after matching are shown in Figures. 2 and 3.

The odds of in-hospital mortality were significantly lower for the HVH group compared with the LVH group (odds ratio [OR], 0.42; 95% confidence interval [CI], 0.22–0.80; P = 0.008) (Table 3).

**Table 3.** Odds ratio of in-hospital mortality for the high-volume hospital group (vs. low-volume hospital group)

**Analysis**	**OR (95% CI)**	**P-value**
Main analysis		
PS matching (n=468)	0.42 (0.22–0.80)	0.008
Sensitivity analysis		
IPTW (n=934)	0.49 (0.29–0.83)	0.009
PS matching excluding patients referred from another hospital (n=232)	0.36 (0.14–0.90)	0.030
PS matching excluding patients who received CPR on day 0^*^ or 1 (n=444)	0.32 (0.13–0.78)	0.012

OR, odds ratio; CI, confidence interval; PS, propensity score; IPTW, inverse probability of treatment weighting; PR, cardiopulmonary resuscitation.

* Day 0 represents the hospital admission day.

### Sensitivity analysis

All prespecified sensitivity analyses demonstrated significantly lower odds of in-hospital mortality in the HVH group than in the LVH group (Table 3).

## Discussion

In this nationwide retrospective observational study, we investigated the relationship between hospital case volume and in-hospital mortality among pediatric patients with sepsis requiring organ support. Our study revealed significantly lower odds of in-hospital mortality for patients treated at HVHs compared to LVHs, addressing a gap in research on this critical issue.

In the present study, the in-hospital mortality rate for pediatric sepsis was 16.1%, comparable to the findings of a nationwide observational study conducted in Germany [24]. Although the mortality rate of pediatric sepsis has been decreasing, it remains a leading cause of death among children in both developing and developed countries [2]. Hence, comprehensive management improvements are necessary to reduce the global burden of sepsis.

A previous study among pediatric patients with infection in the United States demonstrated that those treated in high-volume pediatric intensive care units (PICUs) had lower mortality rates than those in low-volume PICUs [25]. However, >80% of the patients did not have sepsis, leaving the impact of case volume on septic patients uncertain. Our analyses showed a survival benefit for pediatric patients with sepsis requiring organ support measures treated at HVHs, helping to narrow the existing knowledge gap.

The mechanisms underlying the improved survival in HVHs were not explored in this study and warrant further investigation. However, existing literature suggests several potential explanations. First, differences in care process between HVHs and LVHs may exist. A previous study on pediatric sepsis demonstrated that patients with early completion of sepsis bundles showed a lower in-hospital mortality rate [26]. Another study on adult sepsis found that HVHs had better adherence rates to care protocols than LVHs [9]. These findings suggest that HVHs may achieve better patient survival through more consistent implementation of care bundles.

Second, the level of preparedness for treating critically ill pediatric patients may differ between HVHs and LVHs. A study conducted in the United States on critically ill pediatric patients revealed that emergency departments (EDs) with higher weighted pediatric readiness scores (WPRSs) demonstrated superior survival rates compared with those with lower WPRSs [27]. The WPRS assesses the preparedness of EDs for pediatric patients in terms of staff training, staffing levels, equipment availability, and established protocols. The report also showed that high-volume EDs tended to achieve higher WPRSs. These findings suggest that HVHs may have enhanced readiness to manage critically ill pediatric patients compared with LVHs, which could contribute to the higher survival rates of pediatric sepsis cases observed at HVHs in our study.

This study has some limitations. First, the DPC database lacks information on vital signs, laboratory test results, and severity scores (e.g., the Pediatric Index of Mortality 2 or the pediatric sequential organ failure assessment score) [28,29]. To address this limitation, we used organ support measures as surrogate variables for organ failure in the PS model. Second, hospital identification codes in the final dataset were replaced with dummy codes for security reasons. Consequently, we were unable to obtain and adjust for hospital-level information, such as hospital category (e.g., tertiary care emergency hospital or not) and number of PICU beds. Finally, this study did not investigate the effect of hospital case volume on long-term outcomes after hospital discharge.

Nonetheless, our study revealed the survival benefits of treatment at HVHs for pediatric patients with sepsis requiring organ support measures. These findings suggest that several comprehensive measures should be implemented to improve outcomes in this patient population, such as enhancing initial patient management at LVHs and establishing a medical network to facilitate the rapid transfer of critically ill patients to HVHs.

## Conclusion

In conclusion, this study demonstrated that treatment at HVHs was associated with lower odds of in-hospital mortality among pediatric patients with sepsis. Further studies with more detailed patient- and hospital-level information, such as vital signs, laboratory test results, hospital category, and number of PICU beds, are required to elucidate the effect of hospital case volume on mortality.
